# Mass spectrometry data for *in vitro* protein profiles in early and late stages of Douglas-fir xylogenesis

**DOI:** 10.1016/j.dib.2016.03.083

**Published:** 2016-04-01

**Authors:** Jowita A. Dziedzic, Armando G. McDonald

**Affiliations:** a Environmental Science Program, University of Idaho, Moscow, ID, USA; b Renewable Materials Program, Department of Forest, Rangeland and Fire Sciences, University of Idaho, Moscow, ID, USA

## Abstract

A Douglas-fir tissue culture system was developed [1] that could be induced to differentiate into tracheary elements (fibers) making it possible to monitor xylogenesis *in vitro* by a proteomics approach. Two proteomes, one from an early and one from a late stage of fiber differentiation process were analyzed and compared. Obtained mass spectrometry proteomics data have been deposited to the ProteomeXchange Consortium (http://proteomecentral.proteomexchange.org) via the PRIDE partner repository [2] with the dataset identifiers PXD001484 and DOI:10.6019/ PXD001484 [3].

## **Specification Table**

**Table:** 

Subject area	*Biology*
More specific subject area	Tree proteome
Type of data	Raw and processed/analyzed mass spectrometry data
How data was acquired	Mass spectrometry (Reverse phase LC (nanoACQUITY UPLC) coupled to a Q-TOF Premier (Waters) MS/MS system)
Data format	Raw data (.mzML), peak (.mgf), processed and analyzed Mascot search engine (.dat) and result (.dat-pride.xml.gz)
Experimental factors	Non-differentiated and differentiating tissues
Experimental features	Solid and suspension tissue cultures from young Douglas-fir trees were used to initiate *in vitro* xylogenesis. Utilizing 2D SDS PAGE coupled to mass spectrometry, two proteomes were analyzed and compared, one from an early and one from a late stage of the fiber differentiation process.
Data source location	Moscow, ID, USA
Data accessibility	Accessible at ProteomeXchange Consortium via the PRIDE partner repository with the dataset identifiers PXD001484 and DOI:10.6019/ PXD001484.

## **Value of the data**

• This research contributes to the very poorly studied tree proteome and to the gymnosperm proteome, in particular.
• The data presented shows differences in Douglas-fir proteins expressed during cell differentiation using an *in-vitro* culture system.
• The presented *in-vitro* softwood model system has a potential to be used as a basis for future studies involving genetic modifications and as a screening tool for biotechnology programs aiming to improve wood quality.

## 1 Data

Xylogenesis (a process of wood fiber formation) is often characterized by two distinct stages: “early” and “late”. The first stage involves undifferentiated precursor cells that are characterized by their primary cell wall, whereas the later stage includes a succession of events including secondary cell wall deposition and programmed cell death leading to full cellular differentiation into tracheary elements [4]. In this study we aimed to elucidate the differences in proteome composition between those two stages. By means of mass spectrometry analysis we identified significant enrichment in proteins related to cellular energy together with protein and primary cell wall metabolism in undifferentiated samples, whereas differentiated wood fibers were exhibiting peptides involved in cell wall polysaccharide biosynthesis.

## 2 Experimental design, materials and methods

### 2.1 *In-vitro* culture, protein extraction and 2D SDS PAGE analysis

*in vitro* solid and suspension tissue cultures from young Douglas-fir trees were initiated and maintained as described previously [1], [3]. Approximately 4 g of fresh callus was used to inoculate Murashige and Skoog medium supplemented with 3 mg/L BAP, 3 mg/L 2,4-D and 30 g/L sucrose. Subculturing was completed every 10–14 days. To induce tracheary elements formation cultures were maintained for 18 weeks in fresh medium supplemented with 2 mg/L BAP, 2 mg/L 2,4-D and 20 g/L of sucrose.

Phenol-based protein extraction was performed according to the protocol described previously [1]. Protein yields was measured by RCDCTM Protein Assay (Bio-Rad) and analyzed by 2D electrophoresis. Representative 2D SDS-PAGE gels of three replicates were chosen for image analysis. 2D SDS-PAGE image analysis was completed online using LUDESI REDFIN 2D Gel Image Analysis Software (http://www. ludesi.com/software/how-to-use/).

The chosen proteins spots that were present in one image and absent in another (non-differentiated *vs*. differentiating samples) were further analyzed using the MS/MS approach.

### 2.2 Sample preparation and MS/MS analysis

Destained (25 mM ammonium bicarbonate and 50% acetonitrile) and tripsinated (0.5 μM sequencing grade modified trypsin) peptides, were separated using reverse phase LC (nanoACQUITY UPLC) coupled to a Q-TOF Premier (Waters) MS/MS. Protein digests (2 μL) were injected onto a loading column (Symmetry C18 trap column, 180 μm×20 mm) and then analyzed using an analytical column (BEH 130 C18 nanoACQUITY UPLC, 75 mm×150 mm). The Q-TOF mass spectrometer coupled with a nano-electrospray ionization source was used.

The MS and ultra-pressure LC were controlled by Mass-Lynx V4.1 software (Waters). Data generation was performed using a data-dependent analysis (DDA) MS method. When multiple charged analytes (+2, +3, and +4) having properties of peptides were detected, the instrument switched to MS/MS mode. A reference peptide (or lock mass standard, human [glu1]-fibrinopeptide B) was sprayed simultaneously with the LC effluent and was sampled for 1 s every 30 s resulting in a double charged peak at *m*/*z* of 785.826 that was used for a lock mass correction.

### 2.3 Mass spectrometry data analysis

Protein identification and MS analysis was performed by use of ProteinLynx Global Server V2.4 (Waters) The raw data were converted into peak lists (^*^.pkl files) by Protein-Lynx Global Server using the following parameters: (i) smooth channels=4, number of smooths=2, smooth mode=SavitzkyGolay; (ii) percentage of peak height to calculate the centroid spectra=80%; (iii) no baseline subtraction allowed; and (iv) peptide tolerance of 100 ppm. TrEMBL protein database (htpp://www.matrixscience.com) was used to identify amino acid sequenced by cross-species comparison.

The MS proteomics data was deposited at the ProteomeXchange (PX) Consortium [2] via the PRIDE (PRoteomics IDEntifications) partner repository at the European Bioinformatics Institute (http://www.ebi.ac.uk/pride/) and is freely accessible with the dataset identifier PXD001484 and DOI:10.6019/ PXD001484 (htpp://www.matrixscience.com).
