# Supplementary material for: Mass spectrometry data for in vitro protein profiles in early and late stages of Douglas-fir xylogenesis
Source: Data Brief. 2016 Apr 1;7:1048–51. doi: 10.1016/j.dib.2016.03.083 (PMC4927772; doi:10.1016/j.dib.2016.03.083)
Supplement: Supplementary file 1 — Supplementary material [file mmc1.pdf]

# *Conflicts of Interest Statement*

**Manuscript title:** Mass Spectrometry data for in vitro protein profiles in early and late stages of Douglas-fir xylogenesis

The authors whose names are listed immediately below certify that they have NO affiliations with or involvement in any organization or entity with any financial interest (such as honoraria; educational grants; participation in speakers' bureaus; membership, employment, consultancies, stock ownership, or other equity interest; and expert testimony or patent-licensing arrangements), or non-financial interest (such as personal or professional relationships, affiliations, knowledge or beliefs) in the subject matter or materials discussed in this manuscript.

**Author names: Jowita A. Dziedzic and Armando G. McDonald**
**We have no financial conflict of interest**

The authors whose names are listed immediately below report the following details of affiliation or involvement in an organization or entity with a financial or non-financial interest in the subject matter or materials discussed in this manuscript. Please specify the nature of the conflict on a separate sheet of paper if the space below is inadequate.

**Author names: Jowita A. Dziedzic(1) and Armando G. McDonald (1,2)**
**(1)Enviromental Science Program, University of Idaho, Moscow, ID USA,**
**(2)Renewable Materials Program, Department of Forest, Rangeland and Fire Sciences, University of Idaho, Moscow, ID, USA**

**We have no conflict of interest**

This statement is signed by all the authors to indicate agreement that the above information is true and correct (*a photocopy of this form may be used if there are more than 10 authors*):

| Author's name (typed) | Author's signature | Date |
|---|---|---|
| JOWITA A. DZIEDZIC | | 2/18/2016 |
| ARMANDO G. MCDONALD | | 2/18/2016 |
| | | |
| | | |
| | | |
| | | |
| | | |
| | | |
| | | |
| | | |
